# Nutritional Quality of Conventional, Organic, and Hydroponic Tomatoes Commercialized in Quito, Ecuador

**DOI:** 10.3390/foods13091348

**Published:** 2024-04-27

**Authors:** Pamela Y. Vélez-Terreros, David Romero-Estévez, Hugo Navarrete, Gabriela S. Yánez-Jácome

**Affiliations:** 1 Centro de Estudios Aplicados en Química, Pontificia Universidad Católica del Ecuador, Av. 12 de Octubre 1076 y Roca, Quito 170525, Ecuador; pyvelez@puce.edu.ec (P.Y.V.-T.); dfromero@puce.edu.ec (D.R.-E.); 2 Escuela de Ciencias Biológicas, Pontificia Universidad Católica del Ecuador, Quito. Av. 12 de Octubre 1076 y Roca, Quito 170525, Ecuador; hnavarrete@puce.edu.ec

**Keywords:** ascorbic acid, DPPH^+^, lycopene, micronutrients, total phenolics, polyunsaturated fatty acids, trace metals, toxic metals, tomatoes

## Abstract

The consumption of natural foods is increasingly high, and in recent years, consumers have preferred foods from systems with responsible management of natural resources (organic, hydroponic). However, there are still contradictions regarding the nutritional content of products from these different types of crops. Our study aims to compare, for the first time, the content of antioxidants (ascorbic acid, lycopene, total phenolics, essential fatty acids), micronutrients (copper, iron, manganese, zinc), contaminants (cadmium and lead), and free radical scavenging activity between conventional, organic, and hydroponic tomatoes (*Solanum lycopersicum*) sold in markets in Quito, Ecuador. Ascorbic acid and lycopene were determined by HPLC/UV-Vis. Total phenolics (Folin–Ciocalteu method) and free-radical scavenging activity (2,2-diphenyl-1-picrylhydrazyl method) were determined via UV-Vis spectrophotometry. Lipid profiles were determined as fatty acid methyl esters through a GC-FID. Trace metals were determined using FAAS (micronutrients), and GFAAS (pollutants). No significant differences (*p* > 0.05) between antioxidant and micronutrient content among the three types of tomatoes were found. Regarding cadmium and lead, the contents were below the Codex Alimentarius threshold limits. Finally, free radical scavenging activity varied slightly (organic > hydroponic > conventional). Although the samples showed certain differences in antioxidant content, none of the tomato types could be considered nutritionally better because of the high variability of the results.

## 1. Introduction

Present-day nutrition sciences have gone beyond simply determining the nutrient requirements of the human diet to seeking continual improvement of health through the consumption of natural bioactive substances that help to reduce disease risk and deteriorating health [1]. Accordingly, researchers are investigating a new category of health-promoting foods—functional foods and nutraceuticals—to identify and explore these foods’ bioactive substances that provide potential health benefits [1,2]. For example, fruits and vegetables are considered functional foods because they are sources of fiber, vitamins, and minerals [1], as well as bioactive molecules such as polyphenols, flavonoids, and polyunsaturated fatty acids [1,3]. Bioactive substances act as antioxidants to reduce free radicals present in the body [4]. Free radicals and reactive oxygen species (ROS) are unstable and highly reactive compounds [5]. They lead to the propagation of oxidizing chain reactions [5,6] and cause oxidative stress in cells [5,7], affecting cellular structures such as membranes, lipids, proteins, lipoproteins, and deoxyribonucleic acid (DNA). These species also cause apoptosis [8,9] and have been linked to the development and exacerbation of many diseases, including neurodegenerative [10], liver [11], renal, and pulmonary diseases [12]; type 2 diabetes [7]; and atherosclerosis, among others [8,9,13].

In normal human metabolism, the enzymatic antioxidant system, which includes superoxide dismutase, glutathione peroxidase, glutathione reductase, and catalase, may act at different levels, inhibiting the formation of ROS or scavenging free radicals, or increase the antioxidants’ defense enzyme capabilities, preventing their excessive accumulation and consequent adverse effects [7,14]. Other substances, such as ascorbic acid (AA, vitamin C), tocochromanols (vitamin E), carotenoids (pro-vitamin A), selenium, phenolic compounds, flavonoids, and polyunsaturated fatty acids (PUFAs), prevent and reverse the damage done by oxidative stress [7,8,15,16]. Moreover, these substances are effective in reducing disease complications, indicating that they may be beneficial either by ingestion of natural antioxidants or through dietary supplementation [7]. Each of these substances has a different antioxidant capacity (i.e., activity and efficiency) [17]. On its side, AA is a reducing agent that prevents the autoxidation of lipids that generate free radicals in the human body and blocks the oxidation of cell membranes [18]. Lycopene increases levels of antioxidant enzymes such as glutathione reductase activity [19] and protects DNA, lipids, and other macromolecules from oxidative stress [20]. Lycopene also synergizes with other natural compounds, such as vitamin E, which inhibits prostate carcinoma cell proliferation [21] and has demonstrated various health benefits, such as decreasing the risk of chronic diseases like cancer and cardiovascular diseases [13] and performing anti-inflammatory functions in cancer therapy [20,22,23,24]. Total phenolic compounds possess important antioxidant [25,26,27] and anti-inflammatory properties [28], since they neutralize free radicals, break down the process of fat oxidation [29], and restore redox balance to reduce oxidative stress, provide preventive and/or therapeutic effects for cardiovascular disease, neurodegenerative disorders, cancer, and obesity [4]. Finally, PUFAs, mainly α-linolenic and linoleic acids [30], are considered essential fatty acids since humans cannot synthesize them and must consume them in their diets [31,32,33]. These are important for normal human growth and development [31] as they are key components of cell membranes, playing an important role in membrane fluidity and many physiological functions such as blood pressure regulation, cell signaling, and lowering of total blood cholesterol [34].

Fruits and vegetables contain different antioxidant compounds [3,6]. Among these, the tomato (*Solanum lycopersicum* sp.) has been shown to have functional and nutraceutical properties [1,20,22,35] when consumed either fresh or processed [36,37]. Tomatoes are grown worldwide with a compound annual growth rate of 6.7% from 2023 to 2024 [38], as they are tolerant of a wide range of environmental and nutritional conditions [39,40]; indeed, they are the second most widely consumed vegetable, after potatoes [41,42]. In 2022, 1886 hectares of tomatoes were cultivated in Ecuador, producing 52,229 tons [43]. Forty percent of this production came from the province of Chimborazo, followed by the provinces of Pichincha, Tungurahua, Azuay, and Imbabura [44]. Tomatoes are commonly consumed (in salads, and sandwiches, among others), mainly in large cities such as the Metropolitan District of Quito, where these products are available throughout the year [45]. Tomatoes contain many minerals, including macro (major) elements (calcium, potassium, sodium, magnesium), micronutrients (Cu, Mn, Zn, iron (Fe), fluorine, cobalt, chromium, nickel), and toxic trace elements (arsenic, boron, cadmium (Cd), and lead (Pb)) [15]. It also contains vitamins, dietary fiber, protein, essential amino acids, essential fatty acids [15], and bioactive compounds (e.g., phenolic acids, flavonoids, tocopherol, anthocyanins, and carotenoids, including lycopene, β-carotene [13,15,25,26], phytoene, and phytofluene) [22]. Ascorbic acid, lycopene, and phenolic compounds are the main antioxidants found in tomatoes that perform radical scavenging [15,46,47].

Tomatoes’ nutritional composition has been associated with cultivars, harvest time, genetic and agronomic factors, environmental conditions [4,15,41,48,49], and geographic origin [48]. Several studies have been carried out on nutritional differences between organic and conventional tomatoes [48,50,51,52], also related to metal contamination [50,53,54,55]. However, studies on Ecuadorian products are limited, and the results from abroad samples are contradictory [52]; thus, no “best option” between organic or conventional types of crops has been identified [50,56]. Nevertheless, burgeoning awareness of pollutants’ harmful effects on food products—including concerns related to health, lifestyle, the environment, and product quality—has motivated consumers to purchase organic over conventional products [57].

Contrary to farming practices focusing on soil (i.e., conventional vs. organic), hydroponic systems involve growing plants using mineral nutrient solutions in water without soil [58,59]. No information has been found related to the content of nutrients, minerals, or contaminants in Ecuadorian tomatoes of hydroponic crops; thus, differences in their quality remain unclear [60]. Therefore, it is important to empirically evaluate foods’ nutritional content and determine how the beneficial properties of specific types of crops differ to make evidence-based consumption decisions.

To the best of our knowledge, in Ecuador, no previous comparative studies have been carried out related to antioxidant compounds and potential free radical scavenging activity (free RSA) in *Solanum lycopersicum* from conventional, organic, and hydroponic crops. Therefore, our study aimed to quantify the amounts of AA, total phenolics, fatty acids, and lycopene in the three crop types and ascertain their free RSA to evaluate their nutritional benefits. In addition, the quantities of the micronutrients Cu, Fe, Mn, and Zn were determined, as were those of toxic trace metals Cd and Pb, to evaluate compliance with the Codex Alimentarius 193–1995 Standard [61].

## 2. Materials and Methods

### 2.1. Sample Collection and Preparation

Thirty samples of ripened tomatoes ready for consumption (bright red skin, hard texture without bruises) from each conventional, organic, and hydroponic crops were purchased during the wet season (January–March) of 2020 and 2022 from 3 wholesale supermarkets in the Metropolitan District of Quito, Ecuador. A limited number of producers perform organic and hydroponic production in Ecuador. In addition, the organic products sold in wholesale supermarkets must comply with the requirements established by the ISO 17065 standard [62] or the Ecuadorian Agency for Regulation and Control of Phyto and Zoosanitary. Information regarding organic and hydroponic tomatoes is properly indicated on the products’ labels.

Most of the tomatoes sold in the wholesale markets from Quito came from cultivars of nearby cities, mainly from greenhouses in the province of Imbabura (altitude of 1800–2000 m above sea level). The Fortuna hybrid variety is mainly cultivated and then distributed in Quito, as it is a resistant variety and has a longer lifespan [63]. Samples were brought to the Centro de Estudios Aplicados en Química laboratory, where they were washed and rinsed with high-quality reagent water (resistivity 18.2 MΩ cm). Whole samples (as they are generally consumed: with peel and seeds) were subsequently frozen at −20 °C, cut into four pieces, and freeze-dried (Labogene, Bjarkesvej, Denmark) at −50 °C and 0.150 hPa. The freeze-dried samples were ground and stored at −20 °C in amber-colored glass containers to protect them from light until analysis.

### 2.2. Ascorbic Acid Quantification

Ascorbic acid was determined using reversed-phase high-performance liquid chromatography (HPLC). The extraction method was adapted from Chebrolu et al. [64]. Approximately 150 mg of the freeze-dried sample was weighed into amber-colored glass vials, and 4 mL of 3% (*w*/*v*) metaphosphoric acid (analytical grade, Fisher Scientific, Waltham, MA, USA) was added. The mixture was homogenized in a cold (4 °C) ultrasonic bath (40 kHz, 100 W, Branson 2800, Danbury, CT, USA) for 10 min.

For quantification, the modified method from Valle and Rodríguez [65] was used: 10 μL of extract filtered through a 0.45 μm filter was directly injected into the Hitachi LaChrom Elite HPLC system (Tokyo, Japan) with an ultraviolet-visible light (UV-Vis) detector and an Agilent TC C-18 column (150 mm × 4.6 mm, 5 µm, Santa Clara, CA, USA). The chromatographic baseline was achieved in the isocratic mode using a mixture of methanol–water as the mobile phase (15:85 *v*/*v*, HPLC grade, Merck, Darmstadt, Germany), with a pH of 2.5 adjusted with metaphosphoric acid; a flow rate of 0.9 mL·min^−1^; a wavelength of 245 nm; and a total run time of 10 min per sample at 25 °C. Quantification was performed using an external standard (BioXtra, Sigma-Aldrich, St. Louis, MO, USA) prepared in the extraction solution. All samples were analyzed in triplicate, and recovery of the spiked samples was used for quality control. Results are reported in milligrams per 100 g of fresh weight (FW).

### 2.3. Lycopene Quantification

Approximately 125 mg of each freeze-dried sample was mixed in an amber-colored vial with 4 mL of a solution of 15% dichloromethane in hexane (HPLC grade, Merck, Darmstadt, Germany). Samples were homogenized in a cold (4 °C) ultrasonic bath (40 kHz, 100 W, Branson 2800, Danbury, CT, USA) for 10 min. Lycopene quantification was performed according to Tzouganaki et al. [66]: 10 µL of each extract, filtered through a 0.45 μm filter, was injected directly into a Hitachi LaChrom Elite HPLC system (Tokyo, Japan) with a UV-Vis detector and an Agilent TC C-18 column. An isocratic mobile phase of 55/30/15 (*v*/*v*) methanol, acetonitrile, and dichloromethane (HPLC grade, Merck, Darmstadt, Germany) was used. The flow rate was maintained at 1 mL·min^−1^, 30 °C column temperature, and a total run time of 12 min. The analytical wavelength used for lycopene determination was 472 nm, and the quantification was performed using an external standard (analytical standard, Supelco, Sigma-Aldrich, St. Louis, MO, USA). All samples were analyzed in triplicate, and recovery of standard fortifications and spiked samples were used for quality control. Results are reported in milligrams per 100 g of fresh weight (FW).

### 2.4. Total Phenolic Content Quantification

For the extraction of total phenolics, 2 mL of a 19:1 *v*/*v* mixture of methanol (analytical grade, Merck, Darmstadt, Germany) and acetic acid (analytical grade, Mallinckrodt, Staines, UK) was added to approximately 150 mg of freeze-dried sample. The mixture was then homogenized in a cold (4 °C) ultrasonic bath (40 162 kHz, 100 W, Branson 2800, Danbury, CT, USA) for 10 min. Quantification was performed using the Folin–Ciocalteu method [4]. An aliquot of the obtained extract (0.1 mL) was filtered through a 0.45 μm filter and was then added to a mixture of 7 mL of high-quality reagent water and 0.5 mL of 10% aqueous solution of Folin–Ciocalteu reagent (analytical grade, BDH Chemicals, Poole, England) in a 10 mL flask. After 3 min, 1.5 mL of a 20% sodium carbonate solution (analytical grade, Fisher Scientific, East Hampton, NH, USA) was added, and the volume of the mixture was raised to mark with high-quality reagent water. Absorbance was measured at 765 nm using a UV-Vis spectrophotometer (HACH DR/5000U, Ames, IA, USA). Samples were quantified in triplicate using an equivalent calibration curve of gallic acid (for synthesis, Sigma-Aldrich, St. Louis, MO, USA). A gallic acid standard and standard fortifications were used as quality controls. Results are reported in milligrams gallic acid equivalent (GAE) per 100 g of fresh weight (FW).

### 2.5. Total Lipid Content and Fatty Acid Profile Determination

The modified Folch method [67] was applied for the total lipid extraction and lipid profile determination. Approximately 500 mg of the freeze-dried sample was mixed for 10 s with 10 mL of a 2:1 mixture of methylene chloride (analytical grade, Merck, Darmstadt, Germany) and methanol (analytical grade, Merck, Darmstadt, Germany) using an IKA T10 Ultra Turrax homogenizer (Staufen, Germany); this extraction was performed twice. The extract was filtered through a glass filter cup (pore size 4) with a 1 mm Celite^®^ (analytical grade, Sigma-Aldrich, St. Louis, MO, USA) layer and transferred to a 100 mL glass separatory funnel. Subsequently, 5 mL of a 0.73% sodium chloride (NaCl, analytical grade, Merck, Darmstadt, Germany) solution was added and then vigorously hand-shaken for 30 s. The separation was left overnight. The lower phase was collected in a 15 mL amber-colored glass flask, and the solvent was evaporated to dryness at 40 °C using a nitrogen flow (Glas-Col, Terre Haute, IN, USA). The total lipid content (% dry weight) was calculated from the free-solvent weight.

Lipid profiles were determined in triplicate after derivatization to fatty acid methyl esters (FAME). The free-solvent oil was mixed with 4 mL of 0.5 M sodium hydroxide (analytical grade, Merck, Darmstadt, Germany) in a methanol solution; the tube was then heated for 10 min at 90 °C using a water bath. After the tube cooled to room temperature, 5 mL of 20% boron trifluoride in a methanol (Sigma-Aldrich, St. Louis, MO, USA) solution was added and heated for 5 min at 90 °C. When the tube reached approximately 30–40 °C, 2 mL of hexane (chromatography grade, Merck, Darmstadt, Germany) and 3 mL of a supersaturated NaCl solution were added. Finally, the tube was vigorously hand-shaken for 15 s. The organic phase (upper) was carefully collected, filtered through a PTFE 0.45 µm syringe filter, and directly injected into a Perkin Elmer Clarus 500 gas chromatograph with flame ionization detection (Perkin Elmer, Waltham, MA, USA). The analysis was performed using a FAMEWAX capillary column (Restek, Lisses, France, 30 m in length, 0.25 mm internal diameter, 0.20 µm film thickness), with helium as the carrier gas and a flow rate of 1.2 mL·min^−1^; 230 °C and 260 °C were set as the injector and detector temperatures, respectively. The temperature program for the oven was initially set to 100 °C and maintained for 2 min. The temperature ramp was 10 °C·min^−1^ to 140 °C, 3 °C·min^−1^ to 190 °C, and 30 °C·min^−1^ to 260 °C, maintaining the final temperature for 5 min. FAME identification was performed using a commercial mixture standard (37-component FAME mix C4–C24, Sigma-Aldrich, St. Louis, MO, USA). Results are reported as percentages of free-solvent oil.

### 2.6. Free RSA Determination

Approximately 150 mg of the freeze-dried sample was mixed with 2 mL of an 80% acetone solution (analytic grade, Pharmco-Aaper, Toronto, NT, Canada) and homogenized in a room temperature (20 °C) ultrasonic bath (40 162 kHz, 100 W, Branson 2800, Danbury, CT, USA) for 35 min. The extract was then filtered through a PTFE 0.45 µm syringe filter. The free RSA of each extract was measured using the 2,2-diphenyl-1-picrylhydrazyl (DPPH^+^ [4,68,69]). Accordingly, 2 mL of a 0.15 mM solution of DPPH^+^ in methanol was mixed with 0.1 mL of each filtered extract of the sample. The reaction mixture was allowed to react for 15 min in a dark room. Pure ethanol was used as a blank. The absorbances of the DPPH^+^ solution with and without the extract were measured at λ = 514 nm with a HACH-DR/5000U UV-Vis spectrophotometer (Ames, IA, USA).

The free RSA in each sample was determined in duplicate as the percentage decrease in color intensity of the methanolic DPPH^+^ solution [4,69,70], according to the following equation:

(1) $$\%\;RSA=\frac{A_{0}-A_{1}}{A_{0}}\times 100$$

where A_0_ is the absorbance of DPPH^+^ at time 0, and A_1_ is the final absorbance of the samples and standard solution of gallic acid (Merck, Darmstadt, Germany).

### 2.7. Trace Metal Determination

The method described by Romero-Estévez et al. [71] was used to determine trace elements of nutritional interest (Cu, Fe, Mn, and Zn), and toxic trace metals (Cd and Pb). Approximately 500 mg of each freeze-dried sample was weighed in Teflon vials, where 5 mL of 70% nitric acid and 3 mL of 30% hydrogen peroxide (both analytic grade, Fisher Scientific, Waltham, MA, USA) were added. Acid digestion was performed using a MARS 6 microwave (CEM, Matthews, NC, USA). Subsequently, samples were filtered and brought to 50 mL with high-quality reagent water. Cu, Fe, Mn, and Zn levels were determined using an absorption spectrophotometer with air/acetylene flame (AAnalys 400, Perkin Elmer Inc., Waltham, MA, USA), while Cd and Pb contents were determined using an absorption spectrophotometer coupled to a graphite furnace (HGA 900 and AAnalys 400). For quantification, multipoint calibration curves were used. Matrix modifiers for Cd (mixture of 0.015 mg palladium and 0.01 mg magnesium nitrate) and Pb (mixture of 0.2 mg monoammonium phosphate and 0.01 mg magnesium nitrate) were also used (all Inorganic Ventures, Christiansburg, VA, USA). All samples were analyzed in triplicate, and results were recorded in fresh weight (fw). Recovery rates of fortifications and standard controls were used for quality control, as well as DORM-5 (National Research Council Canada, Ottawa, NT, Canada) as a certified reference material. Results are reported in milligrams per kilogram of FW samples.

### 2.8. Calculations and Statistics

The quantification of AA, total phenolics content, lycopene, and trace metals used external standard calibration curves, which were evaluated using the coefficient of determination (*R*^2^ > 0.99). For AA and lycopene, the method limits of detection and quantification were calculated using the variability of 10 blanks by multiplying the standard deviation of the mean blank concentration values by 3 and 10, respectively. For phenols and trace metals, the limits of detection and quantification were determined experimentally. The recovery of standard spiking (AA, lycopene), fortifications (lycopene, phenolics, and Pb), standard control (Fe and Mn), and certified reference material (Cd, Cu, and Zn) was calculated, as were the means and standard deviations of the results. To compare analyte content between the three tomato types, we performed one-way analyses of variance and t-tests, assuming equal variations between each group of samples. Pearson correlations were used to explore associations between analyte and tomato type, as well.

Parametric statistical analyses (*t*-test and ANOVA) were performed using Microsoft Excel 2016 Real Statistics data analysis tools (Microsoft Corporation, Albuquerque, NM, USA) and IBM SPSS Statistics version 28.0.0.0 (IBM, Armonk, NY, USA), in order to identify differences (confidence interval of 95%) between micronutrient and antioxidant content and the three farming techniques of tomatoes.

## 3. Results

### 3.1. Analysis Results

The calibration graphs were performed using an external calibration method. For all methodologies, the *R*^2^ values were >0.99, which suggests a linear fit of the experimental data to the calibration curve (see Table S1).

Regarding the quality control for each assay, the results were within the acceptance limits described in the “Guidelines for Single Laboratory Validation of Chemical Methods for Dietary Supplements and Botanicals” [72]. The quality control recovery rate results and relative standard deviation percentages for the analyte are summarized in Table S2.

### 3.2. Differences in Antioxidants between the Three Tomato Types

In the present study, the AA results differed among tomato types: organic > conventional > hydroponic (Table 1). The hydroponic samples showed the lowest values; however, these results were not significantly different (*p >* 0.05) from the conventional samples. Previous studies have reported variable AA levels in conventional tomatoes. For example, some authors [13,73,74,75,76] found higher values (>20.0 mg·100 g^−1^), while other researchers [3,77,78,79,80] reported lower values (<20.0 mg·100 g^−1^) than the present study. The range of AA results reported in Table 1 was within previously reported ranges (8.40–32.97 mg·100 g^−1^) [26,74,76,81,82,83,84,85,86], including studies on Ecuadorian products (14.6–21.8 mg·100 g^−1^) [87]. Regarding organic tomatoes, Borguini et al. [48] and Vinha et al. [76] found higher AA content than conventional tomatoes, which is consistent with the present study’s results. Rossi et al. [88] found that conventional tomatoes had almost twice the content of AA than organic ones. In the case of hydroponic tomatoes, Arias et al. [89] and Fanasca et al. [90] have reported higher AA contents (20.17 ± 0.40 mg·100 g^−1^ FW and 37.88 mg·100 g^−1^ FW, respectively) than in the present study.

For lycopene (Table 1), no statistical differences were found between the three tomato types (*p* > 0.05), and values were below the range previously reported (4.83–15.18 mg·100 g^−1^) [13,15,86,87,88,91]. However, our results were consistent with other previous studies [3,26,78,80]. Also, organic samples showed similar lycopene content as conventional samples as reported by Borguini et al. [48] and Vélez-Terreros et al. [52]. Rossi et al. [88] reported slightly higher values of lycopene content in conventional tomatoes compared to organic samples. For hydroponic samples, a previous study reported higher lycopene content (>6 mg·100 g^−1^ FW; [89]), but Ajlouni et al. [92] reported similar lycopene content (3.61 ± 0.42 mg·100 g^−1^ FW) to our results.

In the case of total phenolics, the highest content was found in organic tomatoes, followed by conventional and hydroponic (Table 1). All total phenolics values of the tomatoes were lower than those previously reported for both Ecuadorian and non-Ecuadorian tomatoes (>30.00 mg GAE·100 g^−1^ fw) [84,87]. Conversely, for conventional and organic tomatoes, our results were higher than the values reported by other authors [13,80]. Further, in our study, the levels of total phenolics in organic and conventional tomatoes were similar (*p* > 0.05), and both were approximately 1.5 times higher than those of the hydroponic tomatoes. In a study on cherry tomatoes grown organically, total phenolics content was almost twice that found in soilless-grown tomatoes [31], which is in line with our results.

Regarding total lipid content, it was between 2.62% (organic) and 3.20% (hydroponic) of dry weight (Table 1), slightly lower than the previously reported 3.62–5.39% [15]. The most common fatty acids were palmitic (16:0), stearic (18:0), oleic (18:1ω9), linoleic (18:2ω6), and α-linolenic (18:3ω3) acids, which together accounted for approximately 98% of the total fatty acids detected in the three types of tomatoes. It is well known that linolenic is the predominant fatty acid in tomato pulp [32]. Levels of linoleic and α-linolenic acids (Table 2) were consistent with a previous study [15]. Hydroponic samples showed the lowest amount of linoleic acid, and conventional tomatoes had the lowest α-linolenic acid. Thus, conventional, organic, and hydroponic tomatoes had high PUFA quantities (57.38%, 61.06%, and 52.98%, respectively). Contrary to our results, Fernandes et al. [31] found almost twice the amount of PUFAs for hydroponic vs. organic cherry tomatoes. Nevertheless, they also determined that hydroponic cherry tomatoes had the highest levels of monounsaturated fatty acids, similar to our results (Table 2).

### 3.3. Free RSA in the Different Tomato Types (DPPH^+^)

**Figure 1:**
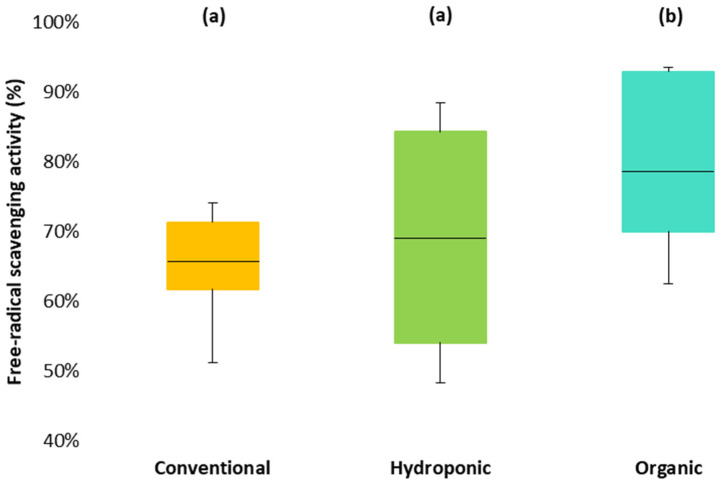
Free radical scavenging activity of the three tomato types. Types of crops with different letters indicate that there was a statistically significant difference between means at the 5% level.

Free RSA refers to the ability of antioxidants in fruit to inhibit oxidation [93] and is related to the substitution of hydroxyl groups attached to phenols’ aromatic rings, which promotes their hydrogen-denoting ability [94]. The results of free RSA varied widely within each tomato type: organic > hydroponic > conventional. However, differences between types were not significant (Figure 1).

Compared to our results, other studies have demonstrated similar values (63.4%) of free RSA for conventional tomato extracts [91], while others slightly lower (54.3%) [13]. Borguini et al. [48] showed relatively higher free RSA levels in organic vs. conventional tomatoes, as the former had a significantly higher amount of AA and total phenolics. Similar results were obtained in the present study, which is consistent with evidence that high lycopene and phenolic content have a noticeable effect on scavenging free radicals [94].

Finally, a strongly significant Pearson correlation was observed within the antioxidant compounds and between these and free RSA (Table 3), confirming free RSA was correlated to both individual antioxidant biomolecules and combined antioxidant compounds.

### 3.4. Trace Metal Content

**Figure 2:**
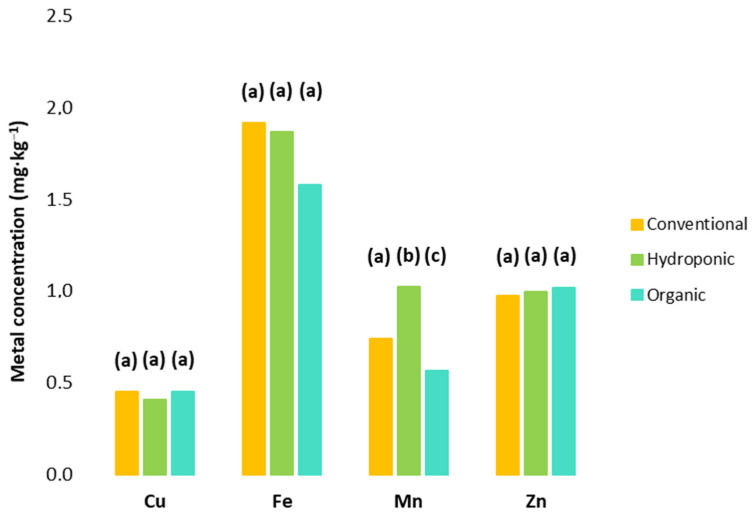
Micronutrient content in conventional, hydroponic, and organic tomatoes. The results from one sample of hydroponic tomatoes were not considered, as these were the highest within the group. Micronutrient metals with different letters indicate that there was a statistically significant difference at the 5% level between each type of crop within each element.

Tomato is a rich source of micronutrients—e.g., Cu, Fe, Mn, and Zn—which are essential for physiological activity and health benefits [95,96]. Previous studies have determined the essential microcontent of trace metals in tomatoes [46,94,96,97,98,99]. In our study, a single sample of hydroponic tomatoes showed the highest levels for all trace metals analyzed (2.97 mg·kg^−1^, 51.53 mg·kg^−1^, 5.54 mg·kg^−1^, and 8.97 mg·kg^−1^ for Cu, Fe, Mn, and Zn, respectively). One specimen from the hydroponic samples was discarded (outlier) due to cross-contamination, which may have occurred from an irregular practice when the nutrient solution was added. For the remaining samples, the results were similar to those reported by Guil-Guerrero and Rebolloso-Fuentes [46]. A significant difference was found for Mn between the three farming techniques (*p* < 0.05) (Figure 2), whose highest value was detected in hydroponic samples. For Cu, Fe, and Zn, no significant differences were found between conventional, organic, and hydroponic tomatoes (*p* > 0.05). The availability of nutrients during plant growth is an important factor that can contribute to the final content of fruits.

Furthermore, no significant differences in contaminant trace metals were found between the tomato types (*p* > 0.05). For Cd, 40% of samples had quantifiable values. Hydroponic and organic tomatoes had similar mean Cd levels, and both were approximately 1.5 times higher than conventional tomatoes (Table 4). Only one hydroponic tomato sample had high levels of Cd (0.0176 mg·kg^−1^ fw). Previous studies comparing Cd content between organic and conventional tomatoes have had mixed results. Bressy et al. [97] found Cd levels almost three times higher in conventional than organic tomatoes (both at the final maturation stage). Hadayat et al. [55] also reported a higher Cd content in conventional vs. organic tomatoes from Florida supermarkets. However, De Souza Araújo et al. [54] found no significant differences between organic and conventional tomatoes.

Regarding Pb content, all samples were below the quantification limit (fw), except for one hydroponic sample (Table 4). Previous studies have reported a wide range of Pb values: lower values (0.005 ± 0.00 mg·kg^−1^) in organic vs. conventional tomatoes (0.008 ± 0.005 mg·kg^−1^) [55], or similar Pb content in both organic and conventional tomatoes [54]. Regarding hydroponic tomatoes, only one sample showed high levels of Pb (0.0269 mg·kg^−1^ fw).

Additionally, we found that Cd and Pb levels for the three tomato types were lower than the Codex Alimentarius [61] limits—0.1 mg·kg^−1^ fw for Cd and Pb. However, it is well known that toxic metals associated with long-term exposure and food intake can affect human health, including gastrointestinal, respiratory, cardiovascular, reproductive, renal, hematopoietic and neurological disorders, carcinogenesis, and oxidative stress [95,100].

## 4. Discussion

According to previous studies, the content of nutrients such as AA, total phenolics, lycopene, and other components can be associated with changes in light conditions during the growth of tomato plants [101], together with other factors such as the variety, growth season, harvest time, and maturity, as well as the irrigation water and nutrient availability [102,103]. Anza et al. [104] reported that variety and season affected all physicochemical parameters measured in tomatoes. Martinez-Valverde et.al [6] also showed different contents of lycopene and phenolic compounds among different tomato varieties. Environmental conditions such as light exposure have also been shown to be favorable factors for the accumulation of nutritional components (such as AA) in tomatoes [102]. Mitsanis et al. [101] discussed that exposure to sunlight promoted the synthesis of AA and total phenolic content, while lycopene synthesis took place in fruit shaded by foliage. As discussed, antioxidant content depends on genetics and is mainly related to the temperature and hours of light present during the growth of the tomatoes [102]. Furthermore, the use of fertilizers rich in soluble nitrogen indirectly and unfavorably could affect the amount of these compounds accumulated due to the increase in leaf density and subsequent decrease in light radiation on the fruits, reducing, for example, the content of AA [41].

The location of Ecuador (in the Equator) produces little seasonality throughout the year [105]. In the particular case of the Andean region of Ecuador―the province of Imbabura, where almost all of the tomatoes sold in Quito are produced―there are only 2 seasons, dry and rainy, and the average temperature differences are not extreme between these seasons—between 8 and 20 °C all year [105]. Therefore, a lower impact of these variables on nutrient content can be assumed, also considering that in all cases, tomatoes are grown in partially or fully controlled conditions within greenhouses.

In the same way, in the Equator, the length of the day changes very little during the year—12 h of sunlight—with variation lower than 30 min at any point in the country [105]; thus, this factor cannot be also considered within the seasonal variability.

Furthermore, as mentioned in the methodology section, in this study, the samples collected came from locations with similar characteristics (Andean region of the Imbabura province). The samples belonged to the same variety (Fortuna) in general and were selected at a visually similar ripening level. Therefore, neither these characteristics nor seasonality were considered variables, allowing us to assume that the type of cultivation technique (organic, hydroponic, and conventional) is the determining factor for the nutritional differentiation of the products that reach consumers.

In our study, AA, lycopene, and total phenolics were higher in organic tomatoes than in conventional and hydroponic ones (Table 1); this higher content of antioxidants is responsible for a higher mean free RSA percentage in the organic (79%) over conventional (66%) and hydroponics (69%) groups (Figure 1). This is attributed to the combination of all the antioxidant molecule activity that increases the fruit extract’s ability to reduce free radicals (DPPH^+^) [106].

Regarding AA, it has been demonstrated that levels in tomatoes increase to a maximum value and then decrease during the ripening process; thus, AA content is lower in tomatoes picked when fully ripe (ready for consumption) compared to those picked at the mature green stage [107].

Fernandes et al. [31] evaluated the nutritional composition of cherry tomatoes grown using hydroponic, semi-hydroponic, and organic techniques. Lycopene was higher in the organic tomatoes (4.71 mg·100 g^−1^), while the hydroponic tomatoes had lower levels (3.58 mg·100 g^−1^), but better texture and taste. Low lycopene content has also been reported by Fanasca et al. [90] in standard tomatoes grown in soilless culture. These low levels might be attributed to climatic conditions inside the greenhouse where the tomatoes were grown, in which the air temperature exceeded 30 °C during the harvest period, leading to a reduction in lycopene biosynthesis [90]. Moreover, it has been reported that higher temperatures during cultivation (above 30 °C) decrease lycopene formation and increase the synthesis of other carotenoids, where fruits become more yellow [108]. Similarly, Caruso et al. [109] examined the influence of two farming systems (conventional and organic) and bio-stimulant application on antioxidant activity and bioactive compound content. Regardless of the bio-stimulant application, conventional farming resulted in better-colored and lycopene-richer fruits, but higher AA content was found in the organic crops. Moreover, the agricultural system also influences the distribution of nutrients in different parts of the fruit. For example, Vinha et al. [76] reported that lycopene was concentrated in the pulp fraction of conventional fruits, while in the organic fruit, the peel and seeds contained high levels of bioactive compounds. In the case of our study, the whole fruit was analyzed, since it is mostly consumed this way.

Regarding total phenolics, previous studies have reported higher polyphenol content in organic vs. conventional tomatoes [76,110,111]; however, phenolics’ levels vary widely and may be affected by ripeness, genotype, and cultivation system [111]. Moreover, field-grown tomatoes have been found to have higher total phenolic content, as light increases the biosynthesis of phenolic compounds in plants by increasing enzyme activity [91]. In a study performed on Greek tomato cultivars, it was found that genetic control is the primary factor determining the content of phenolics, since hydroponic varieties possess antagonistic qualitative traits to commercial tomatoes cultivated in traditional farming conditions [112].

Finally, the ratios of total ω-6/ω-3 acids were from 5.22 in hydroponic tomatoes to 6.56 in conventional tomatoes. According to Chen and Liu [113], the higher the ratio, the more positive PUFAs’ effect on human health, as these compounds can lower low-density lipoprotein and serum cholesterol, reducing cardiovascular disease risk.

A ratio range between 5:1–15:1 for the respective total content of ω-6/ω-3 acids is recommended to reduce cancer and cardiovascular disease risk and enhance bone health [31,113,114]. Our results for conventional, organic, and hydroponic tomatoes fell within this range; however, there is increasing evidence that lowering the ω-6/ω-3 ratio, thus increasing ω3 intake, lowers the presence of other essential fatty acids in the body, especially eicosapentaenoic and docosahexaenoic acid, which directly increase the incidence of chronic diseases characterized by elevated inflammation, including cardiovascular diseases [114,115].

On the other hand, evaluating variation in antioxidant content and free RSA in real time is very complex and also depends on how fruit is stored and transported [48,116]. However, a general approach involves analyzing products commercially available for consumption, which represents what people are truly consuming and was our selected method in the present study.

Pernice et al. [116] showed that particular agricultural practices facilitate the modulation of antioxidant concentration in tomatoes; irrigation and biotype affected flavonoid concentration in fresh and processed tomatoes, while antioxidant activity, irrigation, and biotype were only significant for processed tomatoes.

As with essential nutrients and antioxidants, trace metal content is also influenced by the cultivar, cultivation method, growing conditions, production region, and sampling period [96,98]. It has been reported that tomatoes grown organically contain less Fe than fruits grown hydroponically, which is in line with our results [96]. Additionally, our results for conventional tomatoes showed slightly higher levels of Fe, even if not statistically significant, while hydroponic samples had slightly higher concentrations of Mn (*p* > 0.05). Similar contents of Cu and Zn were determined in the samples of the three types of products (Figure 2). These micronutrients have been recognized as part of the enzymatic antioxidant systems which may remove the ROS and free radicals [7]. There was no significant association between growing methods; nevertheless, essential metals should not exceed concentration threshold values, as they may become extremely toxic in humans [95]. Thereby, in hydroponic growing conditions, the external concentration of nutrients needs to be controlled [117].

Based on our results, Cd levels found in fruits were consistent with those from a previous study [45], which found higher Cd levels in organic tomatoes from “bioferias” markets in Ecuador. Ecuadorian soils are rich in metals such as Cd and Pb, and their presence is mainly attributed to geological processes, such as volcanic activity [95,118]. At the same time, organic agriculture tends to use plant- or animal-based materials such as animal manure or compost to avoid phosphorus fertilizers, which are known to have Cu, Zn, and Cd [119,120]. Unusable waste from plants (leaves, flowers, stems, and roots) is commonly used as fertilizer, and thus, the metal content present in these tissues is reincorporated into the soil and is taken up by the next generation [45]. These aspects of organic agriculture might contribute to higher toxic metal levels in crops.

Pb contamination in crops is not only associated with contaminated soils; its presence in atmospheric particulate matter is related to traffic, road materials, and tires, which in turn could produce post-harvest contamination during crop processing, packing, and transportation [95]. Moreover, in rural areas in Ecuador and different countries worldwide, like China and Egypt, wastewater is used for irrigation, leading to crop contamination [121,122,123]. Ahmed et al. [121] found that Cd was the most accumulated metal, followed by Fe and Pb, in tomato plants from conventional farming.

Even in contaminated soils, solanaceous vegetables like tomatoes have shown the lowest concentrations of Pb, Cd, and As in their edible parts. These trace metals are stored in roots, and their transport to aerial parts of the plant is limited, suggesting they are low accumulators and, thus, suitable for planting in contaminated soils [124]. Piscitelli et al. [125] examined the translocation of Cd and Pb to the edible parts of tomato plants grown in pots with experimentally contaminated soil. Only Cd showed a partial ability to translocate into the aboveground parts of the plants; while Pb was found mainly in the roots, its transport to the aerial parts was extremely limited, and it was absent in the fruit.

The effects of Cd and Pb on tomato plants grown in a controlled hydroponics environment have been also examined. On one hand, López-Millán et al. [126] used different concentrations of Cd solution on tomato plants and found that low Cd concentrations accumulated in roots, but Cd-induced Fe deficiency was observed, whereas high Cd concentrations altered photosynthesis rates, photosynthetic pigment concentrations, chlorophyll fluorescence, and nutrient homeostasis. Azariz et al. [127] determined that Pb accumulates mainly in the roots of cherry tomato plants; a large amount allows it to translocate to the stem and leaves, but only a small amount reaches the fruit.

Furthermore, high levels of toxic metals lead to physiological and biochemical alterations in plants that appear as structural damage and displacement/substitution of essential ions from cellular sites, which affect plant growth, development, and yield [128]. All toxic metals also generate oxidative stress via ROS, which react with DNA, protein, and lipids [129]. Kisa and Öztürk [129] reported that tomato leaves’ exposure to Cd and Pb changed their fatty acid composition, as PUFAs are particularly sensitive to heavy metal stress: linoleic and palmitoleic acid levels were significantly reduced, and the saturated fatty acid content increased. These changes may be related to increased lipid peroxidation due to the presence of ROS, which leads to the fragmentation of PUFA-containing lipids into various products [129].

Toxic trace metals can also affect plants’ primary and secondary metabolite production by changing their molecular, biochemical, and physiological response pathways [130]. Secondary metabolites, such as terpenes, phenolic compounds, flavonoids, and alkaloids, perform diverse functions in plant defense when exposed to metal stress [130]. Kisa et al. [129] reported that while the total phenolics content in tomato leaves decreased under different Cd and Pb exposure concentrations, phenolics levels were highly dependent on growing conditions and stressor presence in fields. They also found that plant responses varied depending on the metal type and metal presence in the plant growth medium [130].

Overall, our results indicated that the Cd and Pb concentrations in all tomato samples were significantly lower than those found before [129,130] (i.e., 10, 20, and 50 ppm); this suggests that metal stress did not influence fatty acid or total phenol content.

## 5. Conclusions

The present study conducted a general comparison of three types of tomatoes (conventional, hydroponic, and organic) to compare nutritional differences between the growing methods. Although certain differences were found regarding antioxidant and micronutrient levels, the wide-ranging results for levels of individual compounds and for the ability to reduce free radicals make it impossible to conclude that any one type is better in terms of quality (i.e., the presence of essential nutrients and antioxidants).

Further, while organic tomatoes had a slightly better nutritional composition, including AA, lycopene, total phenolics, PUFA/SFA, and free RSA, the cultivar, harvest time, genetic and agricultural factors, growing conditions, and production region may directly influence the nutritional and nutraceutical properties of the final product. Moreover, in hydroponic farming, controlling the external concentration of nutrients also has a considerable effect on plants’ nutritional properties.

In the case of contaminants (Cd and Pb), the results were below the Codex Alimentarius maximum limits and even below quantification limits. National regulations to control metal content in crops are still needed, considering Ecuador’s geographic characteristics, natural soil composition, and anthropogenic activities that directly increase nonessential metals’ bioavailability.

The benefits of tomato consumption have been widely studied; furthermore, it has also been proven to have beneficial effects on human health against gastroesophageal reflux disease or heartburn, allergies, kidney and cardiovascular disorders, and prostate cancer. Therefore, it is necessary to carry out further research on long-term tomato consumption and include other substances present in the fruit to accurately assess the related benefits and risks.

## Figures and Tables

**Table 1:** Content of ascorbic acid, lycopene, total phenolics, and main polyunsaturated fatty acids in the different tomato types, mean ± standard deviation.

*n* = 30	Crop Type		
Analyte	Conventional	Hydroponic	Organic
Ascorbic acid (mg·100 g^−1^ FW)	18.52 ± 5.12 ^a^	14.58 ± 6.85 ^a^	23.13 ± 4.43 ^b^
Lycopene (mg·100 g^−1^ FW)	3.27 ± 1.48 ^a^	2.84 ± 1.77 ^a^	3.75 ± 1.71 ^a^
Total phenolics (mgGAE·100 g^−1^ FW)	21.19 ± 4.03 ^a^	14.28 ± 4.59 ^b^	22.99 ± 5.69 ^a^
Total lipids (%)	2.98 ± 0.14 ^a^	3.20 ± 0.34 ^a^	2.62 ± 0.27 ^a^

Values with different letters in the same row indicate that there was a statistically significant difference between means at the 5% level.

**Table 2:** Fatty acid profiles (%) in conventional, organic, and hydroponic tomatoes.

*n* = 30 Fatty Acids (%) x ± s	Conventional	Hydroponic	Organic
SFAs	22.31 ± 0.35 ^a^	23.23 ± 0.25 ^b^	23.94 ± 0.42 ^c^
Miristic (14:0)	n.d.	n.d.	0.19 ^a^
Palmitic (16:0)	16.41 ± 0.30 ^a^	17.13 ± 0.19 ^b^	18.50 ± 0.22 ^c^
Stearic (18:0)	4.48 ± 0.19 ^a^	4.53 ± 0.10 ^b^	3.59 ± 0.04 ^c^
Others ^1^	1.42 ± 0.04 ^a^	1.57 ± 0.05 ^a^	1.66 ± 0.06 ^a^
MUFAs	20.31 ± 0.40 ^a^	23.78 ± 0.14 ^b^	15.00 ± 0.26 ^c^
Palmitoleic (16:1)	n.d.	n.d.	0.25 ± 0.03 ^a^
Oleic (18:1ω9)	20.31 ± 0.40 ^a^	23.78 ± 0.14 ^a^	14.75 ± 0.26 ^a^
PUFAs	57.38 ± 0.50 ^a^	52.98 ± 0.26 ^b^	61.06 ± 0.30 ^c^
Linoleic (18:2ω6)	49.78 ± 0.48 ^a^	44.47 ± 0.16 ^b^	50.80 ± 0.12 ^c^
α-linolenic (18:3ω3)	7.59 ± 0.02 ^a^	8.51 ± 0.11 ^b^	10.26 ± 0.26 ^c^
PUFA/SFA	2.57 ^a^	2.28 ^b^	2.55 ^a^
Σω6/Σω3	6.56 ^a^	5.22 ^b^	4.95 ^c^

n.d.: not detected, SFAs: saturated fatty acids; MUFAs: monounsaturated fatty acids; PUFAs: polyunsaturated fatty acids. The values represent the average of three replicates ± standard deviation. Values with different letters in the same row indicate that there was a statistically significant difference between means at the 5% level. Others ^1^: margaric (17:0); arachidonic (20:0); behenic (22:0); tricosylic (23:0); lignoceric (24:0).

**Table 3:** Pearson correlation between the antioxidant compounds and free radical scavenging activity.

Pearson Correlation (p)	AA	Total Phenolics	Lycopene	Free RSA
AA	1			
Total phenolics	0.878 **	1		
Lycopene	0.604 **	0.528 ** (0.003)	1	
Free-RSA	0.720 **	0.533 ** (0.002)	0.419 * (0.021)	1
Total biomolecules				0.573 **

AA: ascorbic acid; free RSA: free radical scavenging activity. ** The correlation is significant at the 0.01 level (two-tailed). * The correlation is significant at the 0.05 level (two-tailed).

**Table 4:** Results of cadmium and lead quantification in different types of agriculture, mean ± standard deviation.

*n* = 30	Crop Type			Codex Alimentarius ^1^
	Conventional	Hydroponic	Organic	Threshold Value
Cadmium (mg·kg^−1^ FW)	0.0016 ± 0.0007 ^a^	0.0025 ± 0.0054 ^a^	0.0024 ± 0.0021 ^a^	0.1
Lead (mg·kg^−1^ FW)	<0.0200 ^a^	<0.0200–0.0269 ^a^	<0.0200 ^a^	0.1

^1^ FAO/WHO [61]. Values with different letters in the same row indicate that there was a statistically significant difference between means at the 5% level.

## Data Availability

The original contributions presented in the study are included in the article/Supplementary Materials, further inquiries can be directed to the corresponding author.

## Supplementary Materials

The following supporting information can be downloaded at: https://www.mdpi.com/article/10.3390/foods13091348/s1, Table S1. Results of calibration parameters from the determination of ascorbic acid, lycopene, total phenolics, and trace metals. Table S2. Recovery of analytes and relative standard deviation percentage of sample replicates.

## Abbreviations

AA: ascorbic acid; DNA: deoxyribonucleic acid; FAAS: flame atomic absorption spectrophotometry; FAME: fatty acid methyl ester; Free RSA: free radical scavenging activity; GC-FID: gas chromatography with flame ionization detector; GFAAS: graphite furnace atomic absorption spectrophotometry; HPLC: high-performance liquid chromatography; PUFA: polyunsaturated fatty acid; ROS: reactive oxygen species; UV-Vis: Ultraviolet and Visible light.
