# Supplementary material for: Nutritional Quality of Conventional, Organic, and Hydroponic Tomatoes Commercialized in Quito, Ecuador
Source: Foods. 2024 Apr 27;13(9):1348. doi: 10.3390/foods13091348 (PMC11082976; doi:10.3390/foods13091348)
Supplement: Supplementary file 1 [file foods-13-01348-s001.zip › foods-2957244-supplementary.pdf]

Supplementary Table S1. Results of calibration parameters from the determination of ascorbic acid, lycopene, total phenolics, and trace metals.

**Parameter**	**Ascorbic acid (RP-HPLC/UV-Vis)**	**Lycopene (RP-HPLC/UV-Vis)**	**Total phenolic (Folin-Ciocalteu/ UV-Vis)**	**Cadmium (GFAAS)**	**Copper (Flame AAS)**	**Iron (Flame AAS)**	**Lead (GFAAS)**	**Manganese (Flame AAS)**	**Zinc (Flame AAS)**
Detection limit	0.40 ($mg \cdot L^{-1}$)	0.001 ($mg \cdot L^{-1}$)	--*	0.1 ($\mu g \cdot L^{-1}$)**	0.017 ($mg \cdot L^{-1}$)**	0.1 ($mg \cdot L^{-1}$)**	2.0 ($\mu g \cdot L^{-1}$)**	0.033 ($mg \cdot L^{-1}$)**	0.017 ($mg \cdot L^{-1}$)**
Quantification limit	1.34 ($mg \cdot L^{-1}$)	0.29 ($mg \cdot L^{-1}$)	0.0124 ($mg \cdot mL^{-1}$)**	0.5 ($\mu g \cdot L^{-1}$)**	0.05 ($mg \cdot L^{-1}$)**	0.3 ($mg \cdot L^{-1}$)**	5.0 ($\mu g \cdot L^{-1}$)**	0.1 ($mg \cdot L^{-1}$)**	0.05 ($mg \cdot L^{-1}$)**
Linear range	0.5–150 ($mg \cdot L^{-1}$)	0.5–50 ($mg \cdot L^{-1}$)	0.0124–1 ($mg \cdot mL^{-1}$)	0.5–4.0 ($\mu g \cdot L^{-1}$)	0.05–1.5 ($mg \cdot L^{-1}$)	0.3–5.0 ($mg \cdot L^{-1}$)	5.0–40.0 ($\mu g \cdot L^{-1}$)	0.1–1.0 ($mg \cdot L^{-1}$)	0.05–0.7 ($mg \cdot L^{-1}$)
linear equation	$y=263037x-280127$	$y=60509x+41910$	$y=1.29x+0.012$	$y=0.093x+0.0046$	$y=0.126x+0.0010$	$y=0.0160x+0.0004$	$y=0.007x+0.0172$	$y=0.189x+0.0016$	$y=0.561x+0.0199$
R^2	0.9989	0.9981	0.9998	0.9987	0.9996	0.9995	0.9977	0.9998	0.9970

Note: GFAAS: graphite furnace atomic absorption spectrophotometry; flame AAS: flame atomic absorption spectroscopy; RP-HPLC: reversed-phase high-performance liquid chromatography; UV-Vis: ultraviolet-visible light, R^2: coefficient of determination

* The blank value was subtracted from the result.

** Experimentally determined.

Supplementary Table S2. Recovery of analytes and relative standard deviation percentage of sample replicates.

Analyte	Recovery (%)	RSD (%)
Ascorbic acid[1]	89.01–111.01[a]	0.02–8.92
Lycopene[2]	97.54–110.32[a] / 92.38–109.53[b]	0.63–9.84
Total phenolics [3]	94.85–107.58[b]	0.29–5.70
Copper[3]	120.32[c]	< 3.41
Iron[3]	93.86–94.71[d]	0.37–5.66
Manganese[3]	107.50–108.50[d]	0.37–5.90
Zinc[3]	91.89[c]	0.25–2.89
Cadmium[4]	84.13–98.11[c]	< 14.77
Lead[3]	75.08–91.60[b]	< 2.56

[a]spiked samples; [b]fortified samples; [c]certified reference material; [d]standard control
RSD: relative standard deviation.
AOAC (55) limits: [1]recovery 85–110%, RSD: 8%; [2]recovery: 80–115%, RDS: 11%; [3]recovery: 75–120%, RSD: 16%; [4]recovery: 70–125%, RSD: 32%.
